# The Conservative Treatment of the Dental Pulp

**Published:** 1879-05

**Authors:** Richard Grady


AMERICAN JOURNAL

OF

DENTAL SCIENCE.

Vol. XIII. THIRD SERIES—MAY, 1879. No. 1.

### ARTICLE I.

### *The Conservative Treatment of the Dental Pulp.*

BY RICHARD GRADY, D. D. S.

The history of medicine abundantly proves that many of the so-called incurable disorders of the human system are not so in reality, but only apparently beyond the reach of restorative influence for the time being, from imperfect knowledge of their true character, the proper means of relief and the recuperative power of nature. The natural history of disease shows that the most intractable disorders will sometimes resolve spontaneously by the unaided efforts of nature; and, if they do so occasionally without extraneous assistance, is it not reasonable to infer that they would do so more frequently by the aid of enlightened art? Hence in the treatment of all so-called hopeless cases, intelligent and persistent effort should be made not only to relieve immediate suffering, but to correct derangement of every kind and promote complete recovery, giving the patient the benefit of the doubt in every instance, by working steadily for a favorable result even in the most unpromising conditions.

A tendency to conservatism in medicine, surgery and dentistry, is a distinguishing characteristic of the healing art of the present day. The surgeon is no longer ambitious, as formerly, to be distinguished by his brilliant operations. On the contrary his reputation depends upon the intelligence, care and skill with which he labors to preserve and restore members and functions. To preserve the integrity of the physical organism and of the vital forces, and to restore diseased or wounded members whenever possible, may, therefore, be said to be alike the leading principle of surgery, of medicine and of dentistry.

The dentist is constantly called upon to extract teeth for the relief of pain and suffering, which, if he is properly educated and posted in his calling, he is quite certain need not be lost; and which, if he is honest to himself, he will refuse to extract. In operations on the teeth, conservatism may be displayed in a manner to which few give much heed. The dentist is in duty bound, to keep and to guard that portion of the human body which it is his province to treat, as much as the general surgeon is to save to the uttermost that which lies within the province of his art to treat. In fact, there are those in the profession who hold that there is a moral obligation binding upon all men not to mutilate the human body in the slightest degree beyond what is actually demanded as a "dernier ressort," to remove otherwise incurable disease, or in case of extreme suffering, or for the removal of obnoxious deformities.

Let us, then, inquire as to the present status of the profession as regards this sort of practice, how we stand as in relation to the past, and what can be forecast regarding the future, touching some of the prominent points in relation to the subject which seem to demand special attention.

It is comparatively of late years that the pulps of teeth have received anything like conservative treatment from the majority of the dental profession. "*Vis medicatrix naturae*" was not in their dictionary. They had yet to learn the possibilities of nature in her efforts at restoration.

The general practice was to extirpate recently exposed pulps by the surgical or "heroic" operation; and, if this was impracticable, to devitalize them by the use of arsenious acid or the partial luxation of the aching tooth. For many years the essential oils, as sassafras, cloves or cinnamon, were used to devitalize the pulp; in the present day we successfully employ the same essential oils, being guided by knowledge and judgment in their use, for pulp-preservation instead of pulp-devitalization.

Formerly, "some of the most eminent in the profession extracted teeth as soon as the pulps became exposed; some cauterized the surface of the pulps and plugged over them; some used astringents, nut-galls, etc., for an indefinite time and then plugged over them; some plunged a hot instrument into the pulps and then plugged the teeth; others adopted a more refined method, and used the instrument in its cold state; some treated sensitive dentine with the hot instrument. Of course, this was all done, it seemed as if the dental pulp was a sensitive vermicule in a little hole in a bone, and all that was to be done was to kill it with a stunning blow, worry it to death, or smother it by prolonged suffering, or punch it to death with a steel instrument, without the least idea that it was connected with a *living human brain!* and was through that means capable of shocking the whole frame."

When we thus turn back in Dental History, and compare this practice of less than half a century ago with the conservatism of to-day we may reasonably open our eyes in wonder and admiration, and call to mind the old adage of "extremes meeting." In those days it would have been considered absurd, if not quackery, to talk about or advocate the practice now in vogue. Then arsenious acid was looked upon as the most important article in the dental pharmacopœia, because it enabled the dentist to achieve far more in conservative dentistry than any other one thing. But this so-called conservative dentistry meant the preservation of a tooth minus the pulp, "the shell without the kernel."

To-day results not then dreamed of are attainable, and the conservation of the dental pulp is paramount in preserving the teeth. It must not be understood that the writer forgets that the employment of arsenious acid was one of the most important steps taken by the profession, for prior to its use, aching teeth were invariably extracted, as there was no known remedy by which they could be placed in a comfortable condition. Millions upon millions of teeth with exposed pulps have been treated with it and rendered serviceable for years. But, like other useful agents, it has been injudiciously used.

The more the dentist learns about the dental pulp the more convinced does he become that the preservation of the pulps of teeth is an attainment of the first importance, and that it is his duty, as a practitioner of the healing art, to do away with this destructive surgery and make every effort to the conservation of this organ whether in health or disease, because the integrity of the tooth is compromised in every case of destruction, the enamel and dentine becoming friable and portions breaking away. To enable our patients to retain their teeth for a long period of usefulness, should, therefore, be the aim of every member of the profession.

While it is admitted that no class of cases in dental pathology is so hazardous and uncertain in diagnosis and successful treatment as that of the dental pulp, yet it is generally acknowledged that pulps can be saved, that pulps have been saved, and that they are susceptible to the same treatment as other tissues, and when injured must be repaired through a physiological process to restore them to a normal condition. Recognizing the demands of this conservative principle, earnest men in the profession, believing it within the range of possibility, within the unfoldings of dental pathology, within the reach of the persevering student of dentistry, thirty-five years ago, or thereabouts, turned their attention toward devising some method of treatment of exposed pulps which should render it possible to fill such teeth and retain at the same time their vitality.

The advance made in this direction gives great encouragement to hope that the day is not distant when conservative dentistry shall be crowned with the well-deserved praise of carrying healing on its wings.

The pulp tissue is usually intolerant to the presence of foreign bodies; yet nature tolerates a certain amount of effusion. When creasote is applied to the pulp a tissue is formed which is not entirely without vitality, and which may after a time become organized again. Seeds have been preserved alive for hundreds of years; teeth have been transplanted and yet their vitality has been retained; some plants may be dried and kept for an indefinite time and they will revive when moisture is applied to them. How far may this be carried? There is a deposit of cells upon the outside of the living mass of the pulp, and the cells grow until the part is bridged over and the space filled up by their growth extending from point to point of the tooth as the pulp recedes.

When it is recollected that fully nine-tenths of the practice of dentists, apart from mechanical work, involves some treatment either directly or indirectly of the dental pulp, the importance of our subject may be realized: *directly*, the treatment of the pulp, its exposure and its death resulting in alveolar abscess, gives more trouble and anxiety to patient and dental practitioner than any other operation which he is called upon to perform; *indirectly*, even in simple cases of filling when the pulp is not exposed, the dentine through its influence is more or less sensitive.

The capping of a pulp or its extirmination is a small operation in dentistry, but a great one in the saving of a tooth. Sometimes as much skill is required to save a pulp as to save a limb. It is not possible to save all pulps alive, but we may, perhaps, adopt a method of saving many. When pulps are nearly exposed, the dentine may recover its vitality, the salts of lime may be again deposited and thus the pulp be preserved. When the pulp is in a state of suppuration we must consider the condition of the patient, and the result may possibly be dependent on this condition.

Exposure of a pulp in a healthy condition by excavating, is so readily treated and the conditions of the organs so different from those which exist when it is laid bare by disease, that we can hardly draw a comparison between the two cases; yet if we are able to make the latter imitate the former by causing the exposed, ulcerating surface it may be, to assume a healthy one we may surely hope for an equally successful result.

The office of the pulp during a state of healthfulness is not the formation of secondary dentine any more than it is the office of the periodonteum to produce exostosis. Still we know from observed facts, that, under the influence of unusual stimuli, each of these organs is capable of performing this excess of function. Stimulation must, therefore, be moderate, else the primary office of the secreting organ will be impaired through the exhaustion, and normality thereby compromised. Over-stimulation amounts to irritation, and irritation leads to depressed vitality and death.

Surely, then, no one in this enlightened age will deny that a tooth containing a healthy living pulp is of more value than if pulpless; therefore, it is desirable and worth any reasonable effort to preserve the vitality of the pulps of teeth, if it be possible to do so.

In order to appreciate the slightest deviation from health it is necessary to consider what a normal pulp is, and its action in a normal condition. Dental pulps, like all constituents of human bodies, are made up of elements. These consist of four or five arteries and as many veins and nerves held together by connective tissue. Pulps are nourished by pabulum, taken from the blood, which is received through openings in the roots. The pulp diminishes in size with advacing age, the cells covering its surface being converted into dentine. It is not only the mother to the tooth, so to speak, but it is generally recognized as the proper organ through which the tooth receives its nourishment, and therefore, there can be no real safety to the tooth without the pulp in its normal condition; although Mr. Tomes claims that its purpose in a finished tooth is unknown, as also why the tooth should not do just as well without it. In perfect health it may be touched and even incised, (provided it is not pressed) and display little sensibility.

After irritation of any character has continued for a short time, the pulp becomes highly sensitive, and cannot bear contact as it did in a healthy condition. By irritation, then, the pulp is placed in a pathological condition which may lead to the loss of the tooth, if it does receive proper treatment. But with proper treatment, there is no reason why the pulps of teeth, in certain cases to be hereafter named, may not be restored to health as well as other parts of the system having nutrition, circulation and absorption. It is, however, advisable to be thoroughly assured of the life of the pulp; for it would be a less evil to expose a pulp possessed of vitality than to fill a tooth with the decomposed pulp-tissue remaining in it. To determine this question the usual tests, such as percussion, applying streams or jets of hot and cold water, or gentle probing with an excavator in the locality of the pulp chamber, must be resorted to. There must, however, be no uncertainty as to the life or death of the pulp.

Sometimes, in cases of exposure and partial sloughing, a portion of the pulp, included in the fang, presents a normal appearance, except slight fullness in the vessels. In many cases of this kind there is scarcely any soreness or pain, the alveolus being normal and the membrane also, especially in teeth with a single fang. In such cases, is there not an indication that nature is making a saving or conservative effort to retain a portion of such pulp from total annihilation? If so, is not an attempt on the part of the dentist to save not only the tooth but a portion of the pulp also in a healthy condition proper? How long such pulps may live or what per cent. ossify, we have no definite data to decide the point; but we do know that nature proportions her necessities to new conditions.

The writer comes now to consider carefully, and he trusts describe plainly the varieties of pulp-irritation ; the causes to which it is due ; the symptoms by which it is distinguished ; and the modes of treatment to which it is amenable. Dental pulps are irritated by external and internal irritants. J. Tomes thinks that even in as many as 99 cases out of the 100, disease of the pulp as a result of caries is dependent on the perforation of the pulp cavity. Irritation of the pulps of deciduous teeth interferes with the normal absorption of their roots; while irritation of permanent teeth as naturally interferes with the normal formation of their uncompleted roots. Pulps are usually irritated through the medium of external irritants by (1) infiltration of salt, sweet or sour condiments ; (2) direct contact of foreign bodies ; (3) presence of foreign material ; (4) thermal irritation ; (5) mechanical irritation ; (6) medicinally ; (7) prevention of exudation, by filling ; (8) loss of tooth substance by attrition ; (9) fracture of the tooth ; (10) diseases of surrounding parts. These are the external causes from which trouble is liable to arise prior to capping or filling. The cases of frequent exposure or a condition equivalent to exposure are those in which the formative power of the pulp is lowest, the recuperative power least, the progress of decay most rapid, and the consistency of the carious material non-protective.

In considering cavities of decay in relation to pulp cavities, it is necessary to regard (1) situation of cavity of decay, (2) depth of cavity, (3) direction of cavity, (4) character of caries.

In addition to the varieties of pulp-irritation named, there are special cases to be mentioned and considered hereafter. These exceptional cases often occur where the salutary warning which an irritated pulp affords, may be followed rapidly by a disastrous series of symptoms imminently dangerous to the life of the tooth. In considering the different kinds of pulp-irritation it must be remembered that the symptoms to be enumerated as distinguishing each variety, will seldom be all present in any one instance.

There is no routine, strictly so to speak, in affairs of this kind. Every case has more or less its peculiarities; it may be of length of duration, it may be of magnitude, it may be of temperament, it may be of rapidity of progress; and each requires the exercise of educated judgment and practical knowledge. Individual cases alone are of no value, but the accumulation of individual cases leads to the adoption of a theory; and experience teaches that most cases very frequently present the symptoms of more than one variety, according to the influence of the causes to which they are due.

The frequency of pulp-irritation increases in communities as they advance in civilization. We therefore find, as might be expected, that those who chiefly benefit by this advancement, who are in fact the children of civilization, are most subject to diseases of the pulp. Indeed, among the primitive tribes of this country tooth-ache is almost unknown.

When there is extensive decay in a tooth, so that there is no very sound dentine at the bottom down to the pulp, either ossification of the pulp cavity under the decayed region; pulp nodulation, more or less extensive; or complete ossification of the crown portion of the pulp to an indefinite extent down; or death of the pulp, with or without much pain, takes place. The latter result may be attributed to various causes or circumstances, both local and general, depending in some instances on feeble neural resistance, resulting in stasis of circulation, inflammation and death of the pulp. Wedging teeth injudiciously, violent jarring from blows, biting hard substances, and all other causes that disturb the normal condition favor pulp-ossification.

The simple fact that every tooth untouched by disease is possessed of a living pulp, is the best evidence that it should be retained alive, if within the possibilities. We should, therefore, practice conservatism to a radical degree where the dental pulp is involved, for the reason that, after exhausting every resource to save it, it may be deadened and removed as easily as if no effort to save the pulp had been made.

In treating these cases, the first care invariably is to ascertain as far as possible the precise pathological condition which is presented ; whether the pulp is irritated, inflamed, ulcerating or gangrenous. The second point to be considered is the age of the patient; third, the treatment to be adopted; and fourth, the condition of the general health. The pulp may be to all intents and purposes regarding the involvement of its health and life "exposed," while yet there remains enough of original dentinal structure covering it to admit of the careful removal of all decalcified matter in the cavity, without actually coming in contact with it. If this is the case, or if there is actual exposure of recent date, with no great amount of tissue exposed, we may reasonably hope for success; but if, on the other hand, exposure has existed for some time, irritation is extensive and pain therefrom long continued, the suggestions named must be carefully considered.

The age of the patient, as has been stated, will have considerable weight as to the chance of success. In the child and youth, in case of recent exposure, there is reasonable hope of nature's protecting by an extra deposit of bone material, if assisted; while in advanced life, nature may or may not have power enough left to come to the rescue.

The temperament of the patient has of course its bearing upon the results to be attained ; but success or failure does not depend entirely on the habits and temperament. It may as likely depend on the condition of the pulp itself at the time of capping, or, perhaps, the treatment given to it preparatory to capping. The very best temperamental attributes may be rendered almost valueless by an existing state of typhoid, for instance; and, on the contrary, pulps which rank low in the temperamental scale may respond most satisfactorily to treatment, when their possessors are in the enjoyment of excellent physical condition; so that physical condition must be recognized as exerting a most decided influence over the result of every effort at conservation of the pulp. Sex does not seem to affect success in the treatment of pulps, the average of failure being about the same in both cases. It has also been found that the occupation and the surroundings which occupation entails, have a marked influence upon the success or failure of pulp conservation ; as do also the mode of living, place of residence, and thermal and barometric changes.

Every intelligent practitioner will, therefore, readily comprehend that every thing which may possibly influence the final result of such operations, be it climate, temperament, or any thing else which may even in a remote degree become a factor of irritation, should be considered. It will also be necessarily inferred that a quantity of blood, deficient in vital energy or in its constituent properties, and which is, therefore, incapable of healthy coagulation, would thwart the best efforts : hence the various conditions of body must be studied in order to learn as far as may be the limit of possibility in any given case. The predisposing as well as the direct causes of disease must be entertained, and every circumstance carefully weighed according to a judgment well matured by close observation and practice.

Having decided that the pulp can be saved (and Dr. H. L. Sage says in the *Dental Register* : "It is not too much to affirm that at least nine-tenths of the exposed pulps which come into our hands can be protected and saved,") the question arises, How to treat it to that end ?

The proper method of treating an exposed pulp (and by an exposed pulp we do not understand that the pulp is necessarily exposed to view,) has given rise to a vast amount of discussion and difference of opinion. While some gentlemen of acknowledged skill and professional ability claim that, after the pulp has become exposed, inflammation of the periosteum set up and even suppuration of a diseased portion of the pulp commenced, by excising the most diseased portion and using certain therapeutical remedies, such as application of astringents, escharotics and anodynes, followed by filling with some non-conducting material, the diseased pulp can be restored to a healthy condition ; or by capping with gold, lead, tin, horn, oiled-silk, etc., that the vitality of the pulp can be preserved : other gentlemen of equal skill and ability, will invariably devitalize all dental pulps whether recently exposed and in a healthy condition, or when long exposed and in a congested, inflamed or partially dead state.

Now, here are two extremes advocated with an earnestness and zeal, by men of unquestionable veracity and acknowledged ability, and with such force that it seems difficult to come to any just conclusion as to the best method of treatment.

A pulp may not be entirely healthy when exposed by caries, yet there are instances where there seems to be nothing to show that it is in any way diseased. There is little doubt that by proper treatment at the hands of a skillful dentist, a large majority of exposed pulps may be restored to health, and then, if properly capped, the vitality of the tooth can be retained for life. For when a pulp is destroyed by oxy-chloride of zinc, for instance, its death is caused by the caustic effect of the capping at the time of its introduction or immediately following it; and, if not destroyed then, is it any more liable to lose its vitality than a pulp which is not exposed ?

While it is not always, and under all circumstances, advisable to cap exposed pulps, yet when it is desirable, a large majority of teeth so conditioned may be treated and restored to health. How these results can be attained and what remedies are used, are the important questions which we propose to answer.

The operation of capping exposed pulps is by no means new or recent, for we find that Hippocrates is supposed to have borrowed from the Egyptians the practice of cauterizing the pulps of teeth and using cements. Then stimulants, such as alcohol and spirits of camphor, were used for the treatment of cases of exposure of the dental pulp. Later, a gold tube was soldered to the cap so as to allow a discharge from the pulp to pass through the filling without injuring it. Next, collodion with morphia was used ; and, after the first drop flowed over the pulp had set, the cavity was filled with asbestos saturated with collodion.

About 1851, Mr. Tomes advised a mat of gold where the opening is small ; but, if much decomposed dentine exist around the opening, a piece of flexible quill, softened in warm water, should be laid over and the filling completed with amalgam. While Dr. Harris claimed that 11 out of every 12 could be saved by arching over the pulp with gold.

Other methods were suggested, such as lining the inside of the cap with gutta-percha, making a coil of gold wire into a spiral spring for the cap to sustain the filling, asbestos rolled in gold foil, ivory caps, horn, a plate with a hole in it so as to be put in place by an instrument inserted in the hole, excising a portion of the pulp and bringing the edges of the wound into close apposition (thus obtaining healing by first intention and slightly reducing the bulk of the pulp,) bleeding and covering the exposed pulp with oxy-chloride of zinc, creosote and oxide of zinc, dry oxide of zinc, non-irritant cements, cork, celluloid, parchment, gold-beater's skin, letter-paper saturated with carbolic acid for actual contact with the pulp, and oxy-chloride of zinc over this. In addition to the articles already named, there have been recommended for years, oiled-silk and a solution of gutta-percha in chloroform. Of the materials to be placed in immediate contact with the pulp are the additional ones of periosteum, cartilage, skin of boiled egg, (which is very soft and a non-conductor,) balsam of fir. Plaster of Paris is also endorsed because it is plastic, non-irritant, hardens slowly, giving time for careful and easy manipulation ; is alkaline ; porous after consolidation ; and resists sufficiently for plastic fillings, such as oxy-chloride of zinc, gutta-percha or amalgam.

Of the different materials yet used for capping, oxy-chloride of zinc, with its modifications, is claimed to be the

"panacea." It is most in favor for the purpose and offers the much larger proportion of results. It is sufficiently indestructible when protected from the secretions of the mouth. Its conductivity is low, thus preventing disturbance to the pulp by thermal changes. It is dense and hard enough to resist the necessary pressure to complete the operation of filling. It has requisite plasticity to be applied directly upon the denuded pulp, forming a close cap, without crowding upon it. When it can be used without giving pain, it is certainly preferable to any thing in use ; not only for the reasons named but, being a good antiseptic, it prevents the pulp from spontaneous decomposition by coagulating the albumen.

Some eminent dental surgeons aver that the pulp is not saved by the process of capping, but is finally destroyed and taken up by the absorbents, and the canal is left vacant but causing no pain. Granting this to be so in some cases, if the tooth is retained in ease and usefulness, is not the practice commendable and the result attained?

The first step in the treatment of diseased pulps, or in fact any disease, should be to try and find the cause, and if practicable, remove it as a preliminary step ; then the effect may in many instances subside without any further interference. If not, then if the pulp should be coated with creosote, carbolic acid or thymol, a coagulum may be formed sufficiently deep to leave healthy tissue below. Nature having been thus assisted to perform a cure, it but remains to guard the exposed point from liability to repetition of this trouble by placing a safeguard or capping over it and then properly filling the cavity.

When caries has well-nigh laid bare the pulp, it should not be disturbed, but, (after partially cleaning the cavity,) capped with a piece of lead, etc. ; and the cavity filled with plaster, gutta-percha or other soft substance, which should be kept in good condition till the pulp is shielded by new dentine.

The remedies endorsed by Dr. J. Foster Flagg, for pulp-irritation are thus briefly expressed : (1) When the irritation is caused by irritating and escharotic applications, judicious applications and proper protection are the remedies. (2) When caused by excavating; care and accurate knowledge of the pulp cavities are the precautions. (3) When caused by pressure in plugging; lateral pressure, the employment of plastic filling material or the interposition of a solid base is recommended. (4) When caused by conduction or other irritation after plugging; interposition of non-conducting or porous intermediate material is suggested.

*Treatment for Pulp Exposed and probably Aching.* Our first effort is to remove all decay and foreign matter from over and around the pulp without injuring the pulp itself: next, prepare a piece of spunk of suitable size, moistened with carbolic acid, tincture of aconite, or any good anodyne; and, after drying out the cavity carefully, place the spunk over the exposed pulp and seal up the cavity with cotton and sandarac, being cautious not to use too much cotton so as to cause a pressure on the pulp when the cotton swells. Spunk is preferred because it is elastic, yields to protrusion of ossific matter, and prevents contact with the more irritating and unyielding stopping. Let this remain in the tooth, one, two or three days as the case may be, directing the patient to call at the approach of pain. If by this means, an exposed pulp can be kept ten days or even a week without giving any pain, the case may be considered favorable for capping with oxy-chloride of zinc.

If exposure is slight and irritation limited, a thorough bath of pure creosote is generally all-sufficient before capping, yet even here it is safer to move slowly than to cap in haste. In the more complicated cases, careful treatment is of the greatest importance. In every case relief from pain and uneasiness should be secured before capping. To cap a pulp while diseased and irritated, means to destroy its life. The object of medicine is "to entertain" the disease while nature performs the cure. The remedies for this purpose are various and numerous; one proving most successful in the hands of one operator, and another in the hands of another.

But the treatment can not be confined to specifics, though many have appeared from time to time.

*Treatment when Exposed by Excavator.*—If a healthy pulp becomes uncovered and wounded, keep away irritating agents to prevent inflammation, and the pulp will soon heal by first intention, and deposit a sufficient amount of calciferous matter to fill the breach and thus protect itself. If the tooth is aching, apply carbolic acid to allay pain: then after mopping out excess, cover one side of a small cap of paper with a solution of balsam of fir (half drachm of balsam in four drachms of chloroform,) and place it over the wound. The chloroform quickly evaporates and leaves a smooth, glossy coating of soothing balsam which perfectly protects the pulp and holds the paper snugly in its position. Then use oxy-chloride of zinc and complete according to circumstances. But the surest way of saving exposed pulps of this kind is "not to expose them." Pulps not diseased should not be exposed.

*Treatment for Granulating Pulp.*—Suppose the pulp exposed, crimson in color, and even granulating into the carious cavity beyond its normal limits: clean away as much of the softening dentine as possible without inflicting pain; obtund the sensitive surface of the exposed pulp by applying carbolic acid; then lay directly on pulp a piece of card, first immersed in nitric acid. Remove the card in a few minutes, and neutralize the acid in the cavity with an alkali; after which cap the surface with thick paper moistened with carbolic acid, etc. Cover this with a coating of oxy-chloride of zinc, and when this has set, fill with foil, amalgam, etc., according to circumstances.

*Treatment for Sloughing or Pus-Secreting Pulps.*—As to sloughing or pus-secreting pulps few probably are in the habit of treating them with a view of saving and restoring to health the feeble remnant of pulp tissue. When pulps reach this stage, comparatively little is gained by conservative treatment or only in exceptional cases. Still there is treatment for them, and the following is offered: When this condition has been ascertained, first cleanse the carious cavity, removing all trace of decay, and then syringe the suppurating surface of the pulp with carbolized warm water, which soon reveals to what extent the pulp has suffered. Next, cut down the surrounding walls of dentine, so as to be on a level with the surface of the pulp, thus securing the direct apposition of a temporary carbolic acid dressing. The suppurating surface having been changed to a healthy one, apply a bibulous layer securing a strict adaptation in contact with the blanched pulp, when the operation is completed by filling temporarily with os-artificial, or lining the cavity with this material and inserting a good amalgam filling; or, if a gold filling be contemplated, it is wiser to fill with os artificial and defer the gold filling for a reasonable time. This treatment, with certain modifications according to the case, meets all cases of exposed dental pulps, be they healthy, inflamed or suppurating, when this organ has not become irreparably gangrenous or dwindled to dimensions of dead matter only to be met by extirpation and fang filling. It commends itself for: (1) Simplicity and painlessness; (2) Time saving; (3) A great general absence of supervening symptoms; (4) Its wide applicability to all cases of exposed vital dental pulp; and (5) Its *rationale* is sufficiently analagous to the surgical treatment of other lesions of the body.

Topical treatment stands first in the way of removing local irritants, and we have a right to count much upon the recuperative power of the pulp—that *vis medicatrix naturæ* which befriends in the treatment of other lesions of the body, from cuts, splinters under the skin, burns, etc. The peculiar diathesis of the patient may favor or retard the progress of healing, and in so far the local treatment may be seconded by judicious antiphlogistic remedies, aperients and astringent lotions. Constitutional depression of vital power or exhaustion after illness, simply points to the necessity for temporary local expedients; but the principle of the local treatment advocated must not be departed from.

In the methods of treatment of the dental pulp so far noticed, reference has been made to carbolic acid, creosote, styptic colloid, tannin and creosote, and other medicaments of this nature; but it appearing to many a strange way of restoring so delicate an organism as the dental pulp to health, by treating it with strong nitric acid, arsenic, etc., they had recourse to gelatinized phospho-carbonate of lime, which is capable of being rendered plastic for a short time, and hardening very quickly; and lacto-phospho-carbonate of lime. Their advantages are thus briefly expressed. Carbolic acid is not a constituent of dentine, neither is cork or gutta-percha. Nature may tolerate them under certain circumstances, but it is already a forced compliance. When the pulp is exposed it does not ask for any caustic agents, it is already too near destruction. What it does want is bone-material, food that may be converted into ossific matter. In soft, white dental caries, (and it is believed that the dental pulp is only exposed in this kind of caries, 95 per cent. of exposure being made by the excavator or caused by external violence,) if, instead of removing the demineralized dentine that still stands sentry over the pulp, the cavity be dressed with a paste of lacto-phosphate of lime, nature, after a week or more, provides a perfect capping. In using *gelatinized phospho-carbonate of lime* in pulps exposed for some time, mix with the compound, tannic acid and finish at one visit. First saturate with glycerine so that the air may be kept from the pulp while freely exposing it; after removing the decomposed parts, place this lime over the pulp, using pressure till pain is felt, and accelerate the hardening of the lime with absolute alcohol. When hard, treat as in ordinary cases and plug at once.

*Pepsin* has also been recommended for exposed pulps, and of its use, Mr. Oakley Coles says: "Being anxious to find an agent for the treatment of exposed pulps less dangerous in its character than arsenic, and at the same time less painful and fatal to the entire nerve, it occurred to me to try pepsin. Powdered pepsin is mixed into a paste with hydrochloric acid. This paste is left in contact with the pulp and covered with wax for three days; upon removing it the cavity is well washed out with warm water, and swabbed with carbolic acid dissolved in glycerine. The pulp is then capped and the cavity temporarily filled for some months, after which it is filled permanently. The use of the acid is to increase the action of the pepsin, while the glycerine is added simply to preserve the compound in a condition of paste. Believing as I do, in the advantage of preserving the healthy action of a dental pulp, I have found pepsin an exceedingly valuable agent, since it acts only on that part of the pulp which is dead or in an advanced state of disintegration, (as the result of severe inflammation.) The fact that the pepsin paste produces no pain in digesting away the disorganized pulp tissue, is in most instances a still greater recommendation for its use."

When we are exceptionally baffled, and untoward symptoms succeed our efforts to save a pulp, we have an alternative, a valuable expedient in devitalization. Then we must remove all of the pulp that is possible, and "mummyize" by creosote and oxy-chloride of zinc, that all trouble from decomposition and putrefaction is reduced to the minimum. When uncontrollable pulp-irritation demands the devitalization of that organ it may be accomplished, it is said, without the patient experiencing any pain, by applying carbolic acid ten or fifteen minutes previous to the application of arsenical paste.

In conclusion, it is claimed that, although failures do occur in capping pulps, a greater percentage of cases is satisfactory both to the operator and patient when this method is pursued than when the pulps are destroyed and the nerve canals filled; that roots, curved a short distance from the apex, often present themselves that are impossible to fill well, and if not filled to the very end, there is a receptacle left for the retention of irritating matter that will scarcely ever fail to do duty in that direction; and that they are an offset to failures in capping exposed pulps.

We have not facts enough before us to predicate a per centum, but are not the facts already stated sufficient to justify the attempt to save such pulps alive in preference to any other course? And how any progressive dentist can follow exclusively the old method of extirpating pulps and filling canals is hard to comprehend, for really every thing seems to be in favor of making an attempt at capping and preserving these useful organs, although doubtless some pulps die quickly and painlessly after capping.

